# Comparative Outcomes of Limb Salvage Surgery Versus Amputation in Osteosarcoma: A Five-Year Follow-Up Study From a Tertiary Care Center

**DOI:** 10.7759/cureus.94881

**Published:** 2025-10-18

**Authors:** Farhad Ullah, Waheed Altaf, Danish Khan, Noman Alam Khan, Shahid Akbar, Zain Muhammad

**Affiliations:** 1 Department of Orthopaedic Surgery, Lady Reading Hospital MTI Peshawar, Peshawar, PAK; 2 Department of Orthopaedics, Mohi ud din Islamic Teaching Hospital, Mirpur, PAK; 3 Department of Orthopaedics, Lady Reading Hospital MTI Peshawar, Peshawar, PAK

**Keywords:** amputation, limb salvage, osteosarcoma, quality of life, surgical margins, survival analysis, treatment outcome

## Abstract

Background: The most prevalent primary malignant bone tumor that affects teenagers and young adults is osteosarcoma. Limb salvage surgery (LSS) has replaced amputation as the surgical treatment of choice, but still, there is a dearth of comparison data from low- and middle-income environments.

Objective: To compare the outcomes of limb salvage surgery versus amputation in patients with osteosarcoma over five years.

Methodology: The Department of Orthopaedic Surgery at Lady Reading Hospital/MTI, Peshawar, Pakistan, carried out this retrospective comparative study during five years (2018-2023). In all, 61 patients with histologically verified non-metastatic osteosarcoma had either amputation (n=24) or limb salvage surgery (n=37). Data on therapy, histopathology, clinical, and demographics were examined. The EORTC QLQ-C30 questionnaire was used to measure quality of life, and the Musculoskeletal Tumor Society (MSTS) score was used to measure functional outcomes. The Kaplan-Meier method was used to analyze survival.

Results: LSS patients had a significantly higher five-year survival rate (83.8%) compared to amputation (62.5%; p=0.048). Functional scores (MSTS) and quality of life (QLQ-C30) were markedly better in the LSS group (p=0.002). Local recurrence and surgical complications were comparable between groups. Surgical margins were more frequently negative in the LSS group (p=0.031).

Conclusion: Limb salvage surgery suggested a clear advantage in terms of survival, limb function, and quality of life without increasing recurrence or complications. These findings support wider adoption of LSS in appropriately selected osteosarcoma patients, even within resource-constrained healthcare environments.

## Introduction

About 2-3% of pediatric cancers are osteosarcomas, the most frequent primary malignant bone tumor in children and young adults. Its frequency peaks throughout puberty [1, 2]. Around 5 out of every million individuals under the age of 20 receive a diagnosis each year worldwide, and the most common location for tumors is in the metaphyses of long bones like the proximal tibia and distal femur [3]. Limb salvage surgery (LSS), which aims to maintain function and appearance while maintaining oncologic control, has replaced regular amputation as the method of treatment due to advancements in neoadjuvant chemotherapy and surgical reconstruction [4].

The goal of LSS is to remove the tumor without compromising the limb's functionality or aesthetic appeal [5]. It now offers social and psychological benefits in addition to practical ones, making it the preferable option for qualified patients over amputation [6]. However, worries regarding longer recovery times, increased risks of complications, and local recurrence still exist. Amputation, on the other hand, frequently has significant physical and psychological effects, such as phantom limb discomfort, prosthesis reliance, and a lower quality of life, even though it is more definitive in obtaining oncologic clearance [7].

Numerous studies have examined the functional and oncologic results of LSS vs amputation, with differing findings [6, 8]. Some argue that the two strategies have similar survival rates, while others emphasize the higher quality of life and functional scores linked to limb salvage. The majority of the literature that is currently available, however, comes from high-income environments with abundant resources, which may limit its applicability to lower-middle-income nations where patient preferences, infrastructure, and expertise may vary.

Despite a higher likelihood of local recurrence, a 2020 study indicated that LSS was linked to a considerably higher five-year overall survival than amputation; however, there was no significant difference in disease-free survival at five years between the two methods [9]. Similarly, limb salvage surgery independently increased cancer-specific survival (HR ≈ 0.58) compared to amputation, with five-year survival rates of approximately 76.5% vs. 65.1%, respectively, according to a population-based research of adolescents and young people conducted in 2023 using propensity score matching [10].

The evaluation of long-term functional, oncological, and psychosocial outcomes in various therapeutic settings is still necessary, despite worldwide trends that favor limb salvage. By assessing and contrasting the five-year results of limb salvage surgery and amputation in patients with osteosarcoma in a tertiary care facility in Pakistan, where both operations are often carried out, this study sought to close the evidence gap. It will be easier to customize treatment recommendations, enhance patient counseling, and maximize long-term care planning if these outcomes are understood in a setting with limited resources. The goal of the current study was to compare the five-year results of limb salvage surgery and amputation in individuals with osteosarcoma.

## Materials and methods

This five-year observational, comparative, and retrospective study was conducted in the Department of Orthopaedic Surgery and Oncology at Lady Reading Hospital/Medical Teaching Institute (LRH/MTI), Peshawar, the largest tertiary care referral center for bone tumors in Khyber Pakhtunkhwa, Pakistan. The study evaluated patients treated between January 1, 2018, and December 31, 2023, comparing outcomes of limb salvage surgery and amputation for extremity osteosarcoma. Only patients with a minimum of five years of complete follow-up data for survival and functional outcomes were included. All surgical procedures were performed by the same senior orthopedic oncology consultant and his team, following uniform institutional protocols for diagnosis, treatment, and follow-up. The study was conducted in accordance with the ethical standards of the institutional research committee, with approval obtained from the Institutional Research and Ethics Board (IREB) of Lady Reading Hospital (approval number: 168/LRH/MTI; dated December 13, 2017).

The minimum sample size was calculated using OpenEpi version 3.01 (www.OpenEpi.com), with the assumptions of a 95% confidence level, 80% power, and a minimum detectable difference of 20% in five-year survival between the limb salvage and amputation groups. This estimation was supported by previous meta-analyses and clinical studies that demonstrated superior survival outcomes among patients who underwent limb salvage surgery compared with those who underwent amputation [9]. Based on this calculation, 61 patients were included in the study, recruited through non-probability purposive sampling. All consecutive patients treated at LRH/MTI during the study period were screened against eligibility criteria, and those who met them were enrolled.

Patients were eligible if they were between 10 and 50 years of age, had a histologically confirmed diagnosis of non-metastatic extremity osteosarcoma, underwent either limb salvage surgery or amputation as the primary surgical treatment, and had at least five years of complete postoperative follow-up data available. In addition, all patients were required to have received standardized neoadjuvant and/or adjuvant chemotherapy according to institutional protocols. Patients were excluded if they presented with metastatic disease at diagnosis, had recurrent or secondary osteosarcoma, or had incomplete medical records. Exclusion also applied to those without postoperative imaging or follow-up assessments and to patients who were lost to follow-up within the first year after surgery.

Data were collected retrospectively from the surgical oncology unit registry, pathology archives, and the hospital’s electronic medical records. All limb salvage and amputation procedures were performed by the same senior orthopedic oncology consultant and surgical team following uniform institutional treatment and follow-up protocols, ensuring consistency in surgical technique and postoperative management. A standardized data abstraction form was developed and piloted on a subset of cases to ensure consistency. Two investigators independently extracted and entered the data, and any discrepancies were resolved by consensus to ensure accuracy. Information retrieved included demographic characteristics (such as age and sex), tumor characteristics (including site, size, histological subtype, and Enneking stage), and treatment details (including surgical procedure, surgical margins, use of prosthesis or allograft, chemotherapy regimen, and the need for revision surgery). Oncological outcomes were assessed by documenting local recurrence, time to recurrence, metastasis, and survival status at the last follow-up. Functional outcomes were evaluated using the Musculoskeletal Tumor Society (MSTS) scoring system at the most recent follow-up, and quality of life was assessed using the validated EORTC QLQ-C30 questionnaire whenever responses were available [11,12]. Surgical complications such as wound infection, prosthesis failure, delayed union, and re-operations were also recorded.

Patients were followed according to institutional protocols: every three months during the first two years after surgery, every six months during years three to five, and annually thereafter. Follow-up visits consisted of clinical examination, radiographic imaging of the primary tumor site, and chest imaging (X-ray or CT scan) to detect recurrence or metastasis. Functional outcomes and quality-of-life assessments were conducted during scheduled clinic visits or, where necessary, through validated telephone interviews.

Statistical analysis was performed using IBM SPSS Statistics version 26.0 (IBM Corp., Armonk, NY). Continuous variables, such as age, tumor size, MSTS scores, and EORTC QLQ-C30 scores, were reported as means with standard deviations (SD). Categorical variables, including sex, tumor site, histological subtype, surgical margins, local recurrence, metastasis, and survival outcomes, were expressed as frequencies and percentages. Comparisons between the limb salvage and amputation groups were made using the independent samples t-test for continuous variables and the chi-square test or Fisher’s exact test for categorical variables when cell counts were small. Survival outcomes were analyzed using the Kaplan-Meier method, with the log-rank test employed to evaluate differences in survival distributions between groups. A p-value of less than 0.05 was considered statistically significant for all comparisons.

## Results

**Figure 1:**
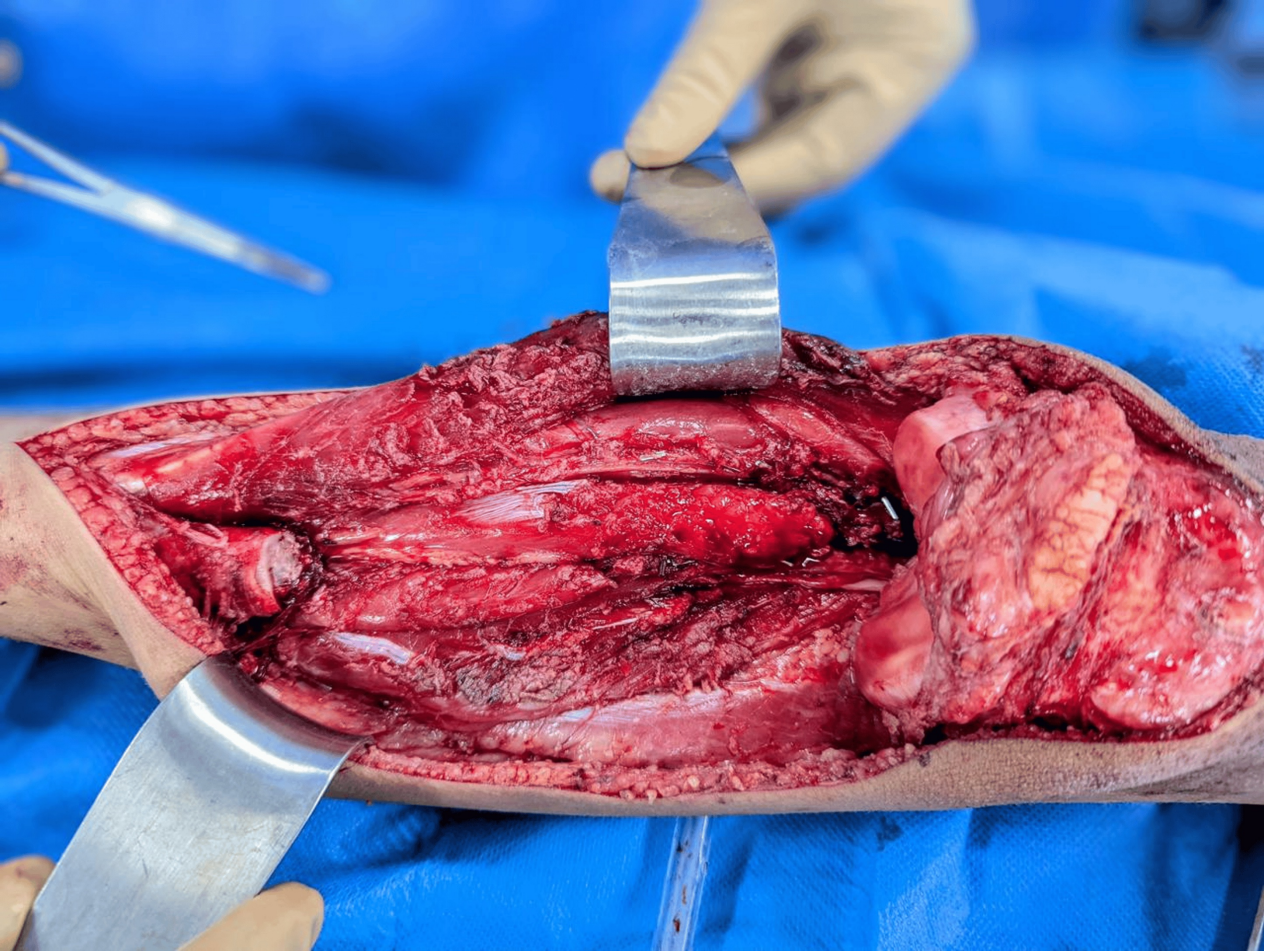
Proximal tibia resection involving osteosarcoma.

The mean age of patients in the limb salvage group was significantly lower than that of the amputation group, based on demographic and clinical characteristics. This indicates that limb-preserving procedures were more frequently performed in younger patients. The gender distribution did not differ significantly between the groups, with a similar male predominance observed in both. The tibia was the most common tumor site in both surgical cohorts, and the overall tumor site distribution was comparable. Representative preoperative and intraoperative images of tibial osteosarcoma and wide resection are presented in Figure 1.

There was no discernible difference in tumor size between the groups, suggesting a similar tumor load at presentation. With the exception of age, these results imply that the baseline tumor features and demographics of the two groups were essentially similar (Table 1).

**Table 1:** Demographic and clinical characteristics of patients (n = 61). A p-value < 0.05 was considered statistically significant. Independent samples t-test was used to compare continuous variables. Chi-square test was applied for categorical variables.

Variable	Limb Salvage (n = 37)	Amputation (n = 24)	Total (n = 61)	p-value
Age (mean ± SD)	20.6 ± 5.2	22.1 ± 6.1	21.2 ± 5.6	0.016
Gender				0.901
Male	22 (59.5%)	15 (62.5%)	37 (60.7%)	
Female	15 (40.5%)	9 (37.5%)	24 (39.3%)	
Tumor Site				0.865
Distal Femur	20 (54.0%)	13 (54.2%)	33 (54.1%)	
Proximal Tibia	11 (29.7%)	6 (25.0%)	17 (27.9%)	
Proximal Humerus	6 (16.2%)	5 (20.8%)	11 (18.0%)	
Tumor Size (cm)	8.4 ± 1.9	9.2 ± 2.1	8.7 ± 2.0	0.325

**Figure 2:**
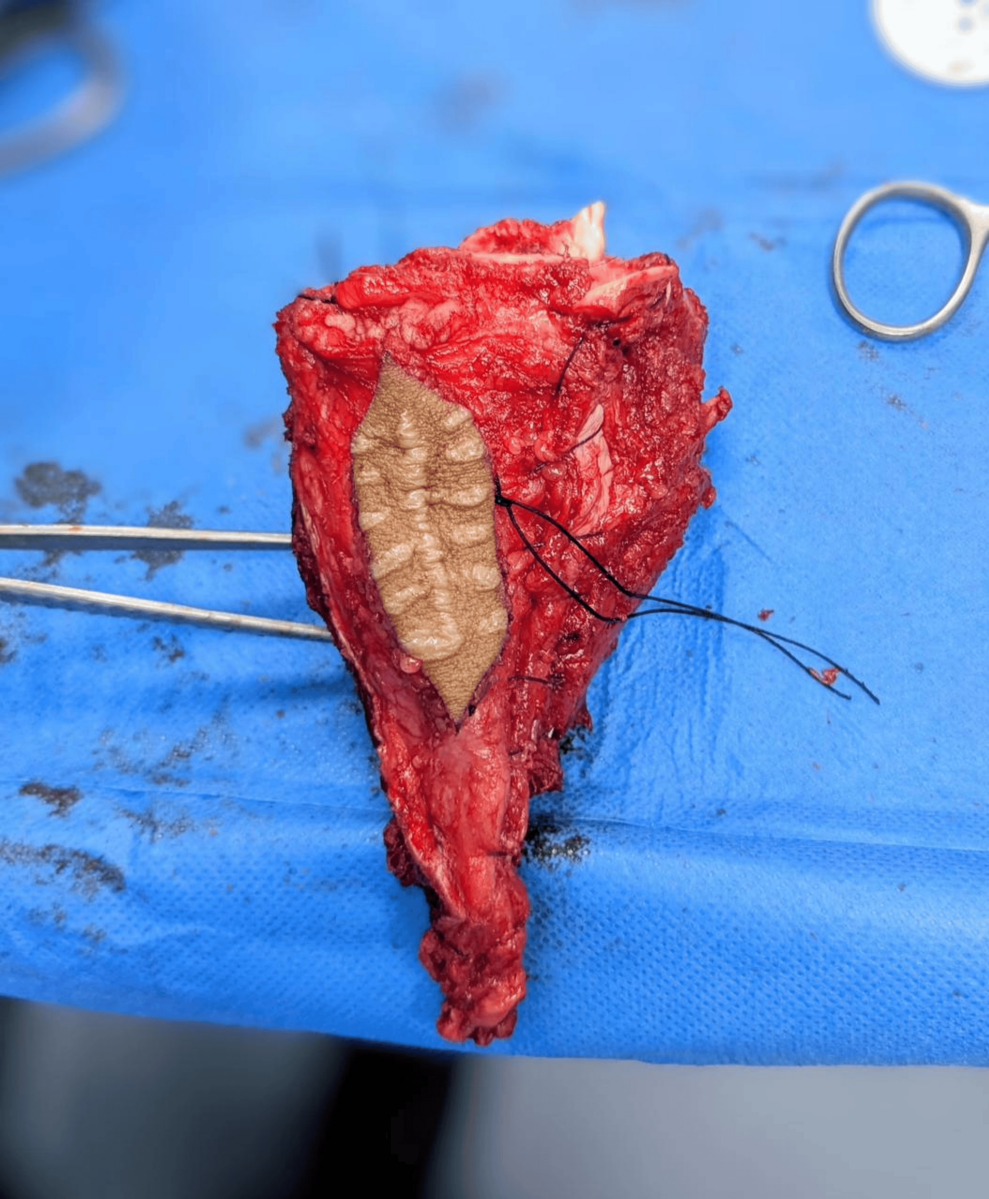
Intraoperative images of wide tumor resection and preparation of resected bone for reconstruction.

Neoadjuvant chemotherapy was administered to every patient in both groups, guaranteeing uniform systemic treatment throughout the cohort. There was no statistically significant difference in the distribution of the histological subtypes (osteoblastic, chondroblastic, and telangiectatic) between the limb salvage and amputation groups. However, the surgical margin status showed a notable variation. Negative margins were more common in patients undergoing limb salvage surgery than in those undergoing amputation, suggesting more thorough tumor resections. An intraoperative view of wide resection and preparation of the resected bone for reconstruction after hydrogen peroxide sterilization is shown in Figure 2. This result emphasizes how well limb salvage works to provide oncologic control in individuals who are carefully chosen (Table 2).

**Table 2:** Treatment characteristics and histopathology findings of the study participants (n=61). *A p-value < 0.05 was considered statistically significant. Chi-square test was applied for comparison of histological subtype. Fisher’s exact test was used for surgical margin status due to small cell counts.

Variable	Limb Salvage (n = 37)	Amputation (n = 24)	Total (n = 61)	p-value
Neoadjuvant chemotherapy	37 (100%)	24 (100%)	61 (100%)	
Histological subtype				0.784
Osteoblastic	25 (67.6%)	17 (70.8%)	42 (68.9%)	
Chondroblastic	7 (18.9%)	3 (12.5%)	10 (16.4%)	
Telangiectatic	5 (13.5%)	4 (16.7%)	9 (14.7%)	
Surgical margin status				0.031*
Negative	36 (97.3%)	19 (79.2%)	55 (90.2%)	
Positive	1 (2.7%)	5 (20.8%)	6 (9.8%)	

**Figure 3:**
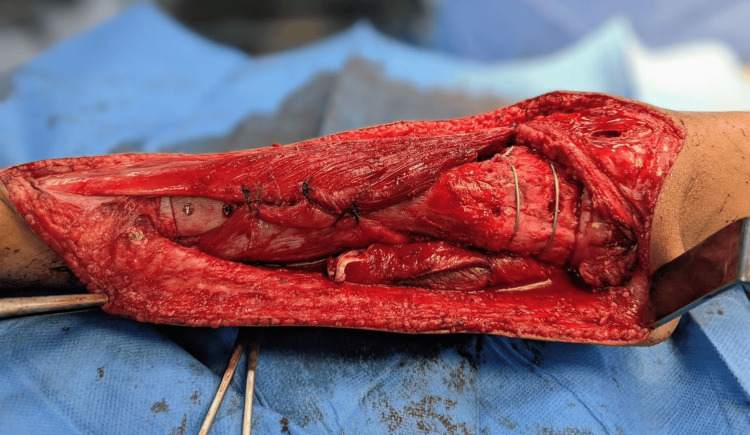
Reimplantation of the sterilized autograft with plate and screw fixation, achieving anatomical restoration and limb preservation after liquid nitrogen sterilization.

With a greater five-year survival rate than the amputation group, postoperative results showed a significant survival advantage for the limb salvage group. Patients who had amputations had a higher incidence of distant metastases; however, this difference was not statistically significant, even though local recurrence rates were modest and almost the same in both groups. Better limb utility and mobility were reflected in the limb salvage group's positive functional outcomes as determined by the MSTS score. Figure 3 illustrates the reimplantation and internal fixation of the sterilized autograft following wide resection, demonstrating successful limb reconstruction and restoration of alignment.

Furthermore, the limb salvage group's quality of life scores, as determined by the EORTC QLQ-C30, were noticeably higher, suggesting improved general well-being. The two groups experienced similar and low rates of surgical complications. Failures connected to the prosthesis were rare and only occurred in the limb salvage group. The Kaplan-Meier method was used for survival analysis, and the log-rank test was used to evaluate the differences in survival distributions between the limb salvage and amputation groups. The limb salvage group had a considerably greater five-year overall survival rate (83.8%) than the amputation group (62.5%). The groups were separated over time by the Kaplan-Meier survival curves. According to the log-rank test, the difference in survival was statistically significant (χ² = 3.91, p = 0.048), suggesting that limb salvage surgery had a survival advantage (Table 3).

**Table 3:** Postoperative outcomes of the study participants (n=61). *Statistically significant (p < 0.05). Chi-square test and Fisher’s exact test were used for categorical variables. The Kaplan-Meier method with the log-rank test was used for survival analysis. Continuous variables were compared using the independent samples t-test. Abbreviations: MSTS = Musculoskeletal Tumor Society Score (out of 30); EORTC QLQ-C30 = Quality of Life score (scale 0–100; higher = better)

Variable	Limb Salvage (n = 37)	Amputation (n = 24)	Total (n = 61)	p-value
Local recurrence	2 (5.4%)	1 (4.2%)	3 (4.9%)	1.000
Distant metastasis	6 (16.2%)	8 (33.3%)	14 (23.0%)	0.132
Five-year survival	31 (83.8%)	15 (62.5%)	46 (75.4%)	0.048*
Functional outcome (MSTS)	26.1 ± 2.4	–	–	
Quality of life (EORTC QLQ-C30)	78.3 ± 5.6	65.4 ± 7.2	–	0.002*
Surgical complications	4 (10.8%)	3 (12.5%)	7 (11.5%)	1.000
Prosthesis-related failure	3 (8.1%)	N/A	3 (4.9%)	–

## Discussion

The results of this study support the growing body of evidence indicating that, compared to amputation, limb salvage surgery (LSS) offers superior functional outcomes, improved quality of life, and favorable long-term survival. In our cohort, local recurrence rates remained low and comparable between groups; however, the five-year overall survival and quality-of-life indicators favored the LSS group [6, 13, 14].

These findings align with recent large-scale analyses. Wang et al. (2022) demonstrated that patients undergoing LSS had significantly higher cancer-specific survival rates compared with those undergoing amputation, with five-year survival rates of approximately 76.5% versus 65.1%, respectively [13]. Similarly, propensity-matched analyses from the National Cancer Database confirmed a survival advantage with LSS, showing a hazard ratio of 1.4-1.7 for amputation-related mortality [15].

Meta-analyses further support these observations. Wang et al. (2023), in an evaluation of adolescent patients, reported that the LSS group exhibited a significantly better five-year overall survival (OR ≈ 4.48) and longer overall and progression-free survival (HR ≈ 0.71), albeit with a slightly increased risk of local recurrence [16]. In a broader meta-analysis, Banskota et al. (2024) concluded that LSS was associated with lower rates of metastasis and higher overall survival, while local recurrence remained similar between treatment groups [6].

Functional outcomes after LSS have consistently been favorable. For instance, in a distal femur cohort (82 patients), prosthesis survivorship reached 67.7% at five years, mean MSTS scores exceeded 26 (≈87%), and recurrence remained modest (~3.7%) [17]. Our findings, demonstrating low local recurrence rates and high MSTS scores, corroborate the viability of modular endoprostheses as durable reconstruction options. However, age-specific differences should be noted. In pediatric patients younger than 10 years, survival outcomes did not differ significantly between LSS and amputation, but LSS was associated with higher complication and reoperation rates [18, 19]. The complication profile in our study, characterized by low prosthesis-related failures and comparable surgical morbidity, reflects this nuance and underscores the importance of patient selection, particularly in younger age groups.

Beyond oncologic control, LSS provides psychosocial and quality-of-life advantages. Historical analyses of Enneking-era and subsequent cohorts indicated that LSS conferred cosmetic and psychosocial benefits without compromising survival, even where functional outcomes were similar to those of amputation [20]. In our study, patients treated with LSS had significantly higher EORTC QLQ-C30 scores, highlighting superior general well-being. Taken together, these results support the notion that, when feasible, LSS offers at least comparable, if not superior, oncologic outcomes, while consistently improving function and quality of life.

Importantly, this study contributes to the literature by presenting data from a tertiary care center in a lower-middle-income setting. Despite potential limitations in follow-up infrastructure, prosthesis availability, and resource allocation, we demonstrated survival and functional benefits consistent with international reports. This reinforces the applicability of LSS in resource-constrained healthcare systems and highlights its relevance beyond high-income contexts. Our findings support the growing consensus that LSS should be considered the preferred treatment strategy for appropriately selected patients with osteosarcoma, even in low- and middle-income countries.

Most notably, our results indicate that limb salvage can be performed safely without compromising oncologic control, provided patients are carefully selected and managed within a multidisciplinary framework. This carries important implications for low- and middle-income countries, where amputation remains common due to historical limitations, and suggests that broader adoption of LSS may improve both clinical outcomes and patient-centered care.

This study has several limitations. First, its retrospective design may introduce biases, including selection and information bias. Second, the relatively small sample size could reduce the generalizability of results and limit statistical power for subgroup analyses. Third, functional and quality-of-life data were available for only a subset of patients, which may underestimate the full variability of outcomes, despite using validated instruments (MSTS and EORTC QLQ-C30). Fourth, while the follow-up period exceeded five years, it may not have been sufficient to capture late complications such as prosthesis wear or secondary malignancies. Finally, as a single-center study, institutional practices and available resources may not reflect broader national or global patterns.

## Conclusions

This study provides compelling evidence that limb salvage surgery provides patients with osteosarcoma with a substantial increase in quality of life, superior functional results, and a considerable survival benefit over amputation, even in a tertiary care setting with limited resources. Our results highlight the clinical viability and long-term advantages of limb preservation when carried out by skilled teams and accompanied by suitable perioperative care, despite the historical reliance on amputation. This study promotes a paradigm shift toward function-preserving strategies that have a significant influence on patient recovery, independence, and well-being while maintaining oncologic safety as surgical oncology develops further. The findings not only support worldwide patterns but also lay the groundwork for regionally tailored treatment plans that take into account local healthcare realities and adhere to international standards.
